# The changing alcohol drinking patterns among older adults show that women are closing the gender gap in more frequent drinking: the Tromsø study, 1994–2016

**DOI:** 10.1186/s13011-021-00376-9

**Published:** 2021-05-26

**Authors:** Line Tegner Stelander, Anne Høye, Jørgen G. Bramness, Geir Selbæk, Linn-Heidi Lunde, Rolf Wynn, Ole Kristian Grønli

**Affiliations:** 1 Division of Mental Health and Substance Abuse, University Hospital of North Norway, Postboks 6124, 9291 Tromsø, Norway; 2 Department of Clinical Medicine, Faculty of Health Sciences, UiT The Arctic University of Norway, Tromsø, Norway; 3 Department of Drug and Tobacco Research, Norwegian Institute of Public Health, Oslo, Norway; 4 Norwegian National Advisory Unit on Ageing and Health, Vestfold Hospital Trust, Tønsberg, Norway; 5 Institute of Clinical Medicine, Faculty of Medicine, University of Oslo, Oslo, Norway; 6 Department of Addiction Medicine, Haukeland University Hospital, Bergen, Norway; 7 Department of Clinical Psychology, University of Bergen, Bergen, Norway

**Keywords:** Alcohol drinking patterns, Alcohol policy, Older adults, Sex differences, Public health, Tromsø study, Norway

## Abstract

**Background:**

As the population of older adults continues to grow, changes in alcohol consumption are important to monitor because an increase may have public health consequences. Rates of alcohol use vary with geographical location. The aim of this study was to examine trends in alcohol consumption among older adults in a geographically defined area in Norway, especially changing sex differences in drinking patterns over a 22-year period.

**Methods:**

Repeated cross-sectional survey (in 1994–95, 2007–08, and 2015–16) of a general population of older adults. Eligible for this study were 20,939 participants (aged 60–99 years). The data were analysed using generalized estimating equations, stratified by age and sex. Alcohol consumption and drinking patterns were assessed, using an adaptation of the AUDIT-C.

**Results:**

Between 1994 and 2016, there has been a significant increase in the proportion of current drinkers among older adults. Furthermore, the probability of frequent drinking (alcohol consumption at least twice weekly) increased significantly between 1994 and 2016, particularly among older women; OR 8.02 (CI 5.97–10.79) and OR 5.87 (CI 4.00–8.63) in the age groups 60–69 and 70+ respectively for women, and OR 4.13 (CI 3.42–4.99) and OR 3.10 (CI 2.41–3.99), in the age groups 60–69 and 70+ respectively for men. The majority of older adults drank small amounts of alcohol on typical drinking days, but there was an increasing probability of drinking three drinks or more on each occasion over the study period, except among women aged 70+ years.

**Conclusions:**

Among older adults in Norway, alcohol consumption in terms of frequency and quantity on typical drinking days has increased considerably from 1996 to 2016. This change is in the opposite direction of what has been reported among younger adults. The gap between women and men in frequent drinking has been markedly narrowed, which indicate that women’s drinking patterns are approaching those of men. This may involve a need to change alcohol policy in Norway to more targeted interventions aimed at older people.

**Supplementary Information:**

The online version contains supplementary material available at 10.1186/s13011-021-00376-9.

Changes in the prevalence of alcohol consumption among older adults may have important public health implications, as alcohol use is a leading risk factor for injuries, mortality and the burden of disease [1–3]. The number of people above the age of 65 is estimated to be doubled by 2050 [4], healthy life expectancy is increasing and the heterogeneity in health status among aging people is greater than in the past [5]. Older adults have a higher incidence of comorbid mental and physical health problems and a higher rate of polypharmacy, compared to younger adults [1, 6–8]. Due to a smaller proportion of body fluids and reduced liver function, which means reduced dose tolerance, older adults are more vulnerable to the physical, psychological and cognitive adverse effects of alcohol, compared to younger adults [9–11]. Older women are even more susceptible than older men, due to naturally lower levels of body water in women than in men, resulting in higher concentrations of alcohol in the blood after drinking equivalent amounts of alcohol [12].

Traditionally, alcohol use has been moderate in older compared to younger adults, and men have had more harmful drinking habits than women, including more frequent drinking and consumption of larger quantities on typical drinking days [13–16]. Recent studies report that older adults in both the Nordic and other European countries have increased their alcohol consumption over the last decades, with diminishing sex differences in drinking patterns [15, 17–21]. However, the size of the changes in alcohol consumption and the size of the changes in differences between the sexes (i.e., prevalence rates of men to women) vary across studies, depending on factors such as social class, ethnicity, and geographical settings [13, 17, 19, 22–24]. Moreover, the prevalence of potentially harmful drinking among older adults varies from 10 to 42%, or even more, as the criteria for “at-risk”, “hazardous” or “unhealthy” drinking in older adults are currently inconsistent and vary between studies [25–28]. The US National Institute on Alcohol and Alcoholism (NIAAA) advises that people older than age 65 who are healthy and who do not take any medicines have no more than seven drinks a week, and no more than one drink on any 1 day, whereas The UK alcohol guidelines of 14 units a week may be to be too generous for older people [29]. Knowledge of the lower limits of potential harm from alcohol is constantly growing, but most countries in Europe, including Norway, lack specific guidelines addressed to older adults [14, 30]. Inconsistency in findings implies possible differences between countries in drinking patterns of older adults and that the importance of sex for drinking patterns might differ between countries. However, the European definitions of “one unit of alcohol” vary between 8 and 20 g of pure ethanol [2], which means that even well-defined guidelines can be interpreted very differently. Longitudinal surveys from different geographical locations are hence needed to investigate variations and monitor changes in alcohol use in different aging populations. In Norway, one unit of alcohol is defined as 12 g of ethanol.

The aim of the present study was to investigate trends in alcohol consumption among older adults (defined as those aged 60 years and over) in an urban municipality in Norway, by comparing participants in a population study from the same geographical setting across 22 years. We aimed to describe age- and sex-stratified changes in i) proportion of current drinkers ii) alcohol drinking pattern in terms of past year drinking frequency, and quantity on typical drinking days (≤2 units/≤24 g of ethanol, here defined as “moderate” or ≥ 3 units/≥36 g of ethanol, here defined as “at-risk”), and iii) heavy episodic drinking (HED) last year (≥6 units/≥72 g of ethanol in one occasion). In particular, we aimed to investigate whether sex differences in alcohol consumption among older adults have changed.

## Methods

### Study design and study sample

Our study design is a repeated cross-sectional examination of a large general population living in a geographically defined area in Norway. The data used in this study are taken from The Tromsø Study, an ongoing population-based cohort study conducted in the municipality of Tromsø, the seventh largest city in Norway. The study was initiated in 1974 and currently consists of seven surveys [31, 32]. A total of 45,473 persons have participated in at least one of the surveys. The present study is based on three of the Tromsø surveys, Tromsø 4 (1994–95), Tromsø 6 (2007–08) and Tromsø 7 (2015–16), in order to examine trends in drinking patterns over the last 22 years. Data were retrieved from participants aged 60 years and over at the time of participation and who answered questions about alcohol consumption. All residents of Tromsø municipality aged 60 years and over were invited to these three surveys, and it thus constituted a random sample. In 1994, the number of inhabitants in Tromsø was 54,600, and in 2016 it had increased to 73,480. Eligible for this study were 5861 participants (55% women) from Tromsø 4, 6462 participants (53% women) from Tromsø 6 and 8616 participants (52% women) from Tromsø 7 (Table 1).

**Table 1:** Overall sample characteristics (≥60 years, *N* = 20,939)^a^

	Tromsø 4 (1994-95)	Tromsø 6 (2007-08)			Tromsø 7 (2015-16)		Total
Men	N (%)	Attendance (%)	N (%)	Attendance (%)	N (%)	Attendance (%)	N
Age groups							
60-69	1,479	87	1,995	74	2,502	71	5,976
70+	1,134	70	1,037	61	1,663	63	3,834
Total	2,613	78	3,032	69	4,165	68	9,810
Age, mean (SD)	69.1 (6.8)		67.8 (6.4)		68.8 (6.7)		
Education (%)							
Higher (>12 years)	360 (14)		980 (33)		1644 (41)		
Relationship status (%)							
Spouse or partner	1819 (82)		2441 (82)		3251 (81)		
Women	N (%)	Attendance (%)	N (%)	Attendance (%)	N (%)	Attendance (%)	N
Age groups							
60-69	1,620	90	2,107	80	2,677	75	6,404
70+	1,628	67	1,323	58	1,774	55	4,725
Total	3,248	76	3,430	70	4,451	65	11,129
Age, mean (SD)	70.5 (7.1)		68.6 (7.0)		68.9 (7.0)		
Education (%)							
Higher (>12 years)	210 (7)		711 (21)		1431 (33)		
Relationship status (%)							
Spouse or partner	1369 (57)		1958 (60)		2654 (65)		

^a^Number of participants (N) and attendance rates of the overall invited residents in Tromsø (%), stratified by age and sex in three surveys from the Tromsø Study

### Measures

#### Alcohol consumption

Alcohol consumption was measured with an adaptation of the AUDIT-C (Alcohol Use Disorders Identification Test-Consumption), which is an abbreviated version of the 10-item AUDIT [33], consisting of three items on the past years` frequency of drinking (never, monthly or less, 2–4 times a month, 2–3 times a week, or 4 or more times a week), number of drinks on a typical drinking day (1–2, 3–4, 5–6, 7–9, or 10 or more), and frequency of heavy episodic drinking (HED), 6 units or more (≥72 g of ethanol) in one sitting (never, less than monthly, monthly, weekly, daily or almost daily). The AUDIT-C is recommended for identifying at-risk drinking prevalence in older adults [28]. We dichotomized drinking frequency to “infrequent” (< 2 times a week) or “frequent” (≥2–3 times per week) drinking, as this cut-off limit is used in other comparable studies [7, 34]. Due to some evidence on cut-off limits of at-risk drinking among older adults [26, 35, 36], we dichotomized drinking quantity to “moderate” (≤2 units/≤24 g of ethanol) or “at-risk” (≥3 units/≥36 g of ethanol) drinking on typical drinking days. HED was dichotomised to “never” or “ever”, due to the fact that HED at least once yearly identifies those at risk of harm from any heavy drinking [28, 33].

The questionnaires on alcohol consumption differed slightly in Tromsø 4. Abstinence was measured by the question; “Are you a teetotaller” with response alternatives “yes” or “no”. Frequency was measured by an open question: “During the last month, how often did you consume alcohol?”. Quantity was measured by the question; “How many drinks of beer, wine and spirits do you consume during a usual two-week period?”. The question about HED was the same in all three surveys, but was asked only to participants < 70 years in Tromsø 4. Supplementary Table 1, Additional file 1, gives a comprehensive description of the measurements of alcohol consumption in Tromsø 4 and how they were operationalized to be comparable to the measurements in Tromsø 6 and 7.

#### Sociodemographic variables

Age was measured as a continuous variable and subsequently recoded into two age groups: 60–69 years, and 70 years and older (70–99). Sex was coded 0 (females) and 1 (males). One questions about educational level was included. In Tromsø 4 and 6, there were five response categories; 1) 7–10 years primary/secondary school 2) Technical school, middle school, 1–2 years senior high school 3) High school diploma (3–4 years) 4) College/university, < 4 years, 5) College/university, ≥4 years. In Tromsø 7, there were four response categories; 1) Primary/partly secondary education (up to 10 years of schooling) 2) Upper secondary education (a minimum of 3 years) 3) Tertiary education, short (college/university, < 4 years) 4) Tertiary education, long (college/university, ≥4 years). We dichotomized educational level into 1) Lower educational level (categories 1–3 in Tromsø 4 and 6, and categories 1–2 in Tromsø 7), and 2) Higher educational level (categories 4–5 in Tromsø 4 and 6, and categories 3–4 in Tromsø 7). One question about living situation was included: “Do you live with a spouse/partner?” with two response alternatives: “yes” or “no”.

#### Statistics

Continuous variables are presented as the mean (SD) and categorical variables as counts (%). Prevalence rates, sex differences and changes in sex differences in abstaining, infrequent/frequent drinking, moderate/at-risk drinking, and any/none HED last year were calculated for the total sample and separately for the age groups 60–69 and 70 + .

Since a number of the individuals in this study participated in two (Tromsø 4/Tromsø 6 = 1589; Tromsø 4/Tromsø 7 = 583; Tromsø 6/Tromsø 7 = 3975) or all three of the surveys (545), these observations are considered clustered or non-independent. To account for this dependency, we used generalized estimating equations (GEE) for fitting logistic regression models. We specified models, with a logit link function, the correlation structure was set to exchangeable, and we selected robust standard errors. Binary variables of abstainers/drinkers, infrequent/frequent drinkers, moderate/at-risk drinkers and any/not HED last year were compared across time. Time (1994–95, 2007–08 and 2015–16) was used as an independent variable. 1994–95 was set as reference category in all models, except for HED in age group 70+. The question about HED was asked only to participants aged < 70 years in 1994–95. 2007–08 was thus set as a reference category in the model of older adults 70+, to enable comparison of changes in prevalence and sex differences among participants over 70 years between 2007-08 and 2015–16 in this drinking category. In order to test for changing sex differences between surveys we included an interaction term between sex and survey.

To describe overall changes in drinking patterns in the population of older adults we used unadjusted models. However, age, educational level and relationship status may account for some of the sex differences and in alcohol consumption [17, 19, 23], so these variables were included in the models of change in sex differences. Furthermore, the change in education level and relationship status differed between the sexes during the study period, separate models were therefore estimated to compare the influence of these covariates. Participants reporting to be abstainers were only included in the category of overall drinking/abstaining, and excluded from analyses of other drinking patterns. The results are reported as odds ratios (OR) with 95% confidence intervals (95% CI).

Changes in educational level and relationship status across time among men and women were compared with Chi-square tests. Data were analysed using IBM SPSS (Statistical Package for the Social Sciences), version 26.

## Results

### Sample characteristics

Mean age of the included older adults was 69.9 (SD 7.0), 68.2 (SD 6.7), and 68.9 (SD 6.9) years in the three consecutive surveys (*N* = 20,939). The overall attendance rates among those aged 60 years and over decreased for each survey, from 77 to 69% and 66% in the latest survey. In 1994–95, 69% of participants lived with a partner, compared to 73% in 2015–16. The difference in relationship status was significant among women (*p* < 0.001) but not among men (*p* = 0.421). A proportion of 10% had completed college/university education in 1994–95, compared to 27% in 2007–08 and 37% in 2015–16. The difference in educational level was significant in both women and men (*p* < 0.001 for both sexes).

### Trends in abstaining (full sample)

The overall prevalence rates of abstaining decreased significantly for each of the three surveys from 31% in 1994–95 to 17% in 2007–08 and 11% in 2015–16 (p < 0.001). The prevalence decreased significantly in both men and women and in all age groups during the study period (Table 2).

**Table 2:** Prevalence^a^ of abstaining and drinking patterns (frequency) and odds ratios (OR)^b^ across time

Age at participation	Time^c^	Abstaining (full sample)		Infrequent drinking < 2 times per week (drinkers only)		Frequent drinking ≥2–3 times per week (drinkers only)	
		% (N)	OR (95% CI)	% (N)	OR (95% CI)	% (N)	OR (95% CI)
**Women**							
60–69	1	31.6 (511/1616)	1	94.8 (1048/1105)	1	5.2 (57/1105)	1
	2	15.5 (321/2076)	0.42 (0.36–0.50)	73.9 (1297/1755)	0.18 (0.13–0.24)	26.1 (458/1755)	5.62 (4.16–7.59)
	3	9.1 (241/2657)	0.25 (0.20–0.29)	65.7 (1587/2416)	0.13 (0.09–0.17)	34.3 (829/2416)	8.02 (5.97–10.79)
70+	1	48.2 (779/1616)	1	95.7 (801/837)	1	4.3 (36/837)	1
	2	35.2 (438/1243)	0.64 (0.54–0.76)	82.9 (667/805)	0.26 (0.18–0.39)	17.1 (138/805)	3.80 (2.55–5.66)
	3	23.0 (398/1734)	0.36 (0.31–0.43)	73.1 (976/1336)	0.17 (0.12–0.25)	26.9 (360/1336)	5.87 (4.00–8.63)
**Men**							
60–69	1	14.5 (214/1477)	1	85.8 (1084/1263)	1	14.2 (179/1263)	1
	2	6.4 (126/1974)	0.43 (0.34–0.56)	69.3 (1280/1848)	0.38 (0.31–0.46)	30.7 (568/1848)	2.62 (2.16–3.19)
	3	5.1 (127/2491)	0.35 (0.27–0.44)	58.6 (1385/2364)	0.24 (0.20–0.29)	41.4 (979/2364)	4.13 (3.42–4.99)
70+	1	25.0 (282/1129)	1	87.7 (743/847)	1	12.3 (104/847)	1
	2	18.8 (190/1008)	0.75 (0.59–0.95)	78.5 (642/818)	0.59 (0.45–0.78)	21.5 (176/818)	1.70 (1.29–2.25)
	3	10.8 (179/1644)	0.40 (0.32–0.50)	66.4 (974/1466)	0.32 (0.25–0.42)	33.6 (492/1466)	3.10 (2.41–3.99)

^a^All age group by sex prevalence rate changes were statistically significant between 1994 and 95 and 2015–16

^b^OR from Generalized Equations Models with 1994–95 as reference, stratified by age group and sex, adjusted by educational level and relationship status

^c^Time: 1 = Baseline, 1994–95, 2 = 2007–08, 3 = 2015–16

In the youngest age group (60–69 years), 95% of men and 91% of women reported being current drinkers in 2015–16, compared to 85 and 68% respectively in 1994–95. Results from crude data are shown in Additional Fig. 1, Additional file 2.

**Fig. 1:**
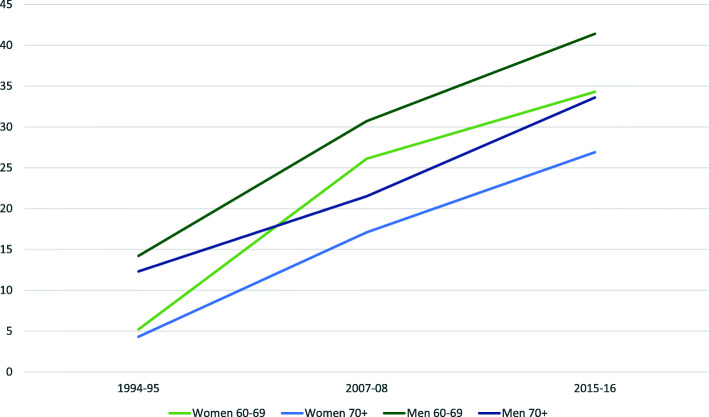
Change in overall prevalence (%) of current drinkers reporting frequent^1^ drinking across time. ^1^Frequent drinking = AUDIT item 1, current drinkers who report to drink 2–3 times per week or more often, stratified by sex and age group

### Trends in alcohol consumption, frequency (current drinkers)

The majority of both men and women reported alcohol consumption once a month or less or 2–4 times per month, in both women and men. However, the prevalence of infrequent drinking was considerably reduced during the study period in all age groups (Table 2). Correspondingly, the overall prevalence of frequent drinking (drinking at least twice weekly) increased significantly for each of the three surveys from 9% in 1994–95 to 25% in 2007–08 and 35% in 2015–16 (*p* < 0.001). The change in sex- and age-stratified prevalence is shown in Fig. 1.

**Fig. 2:**
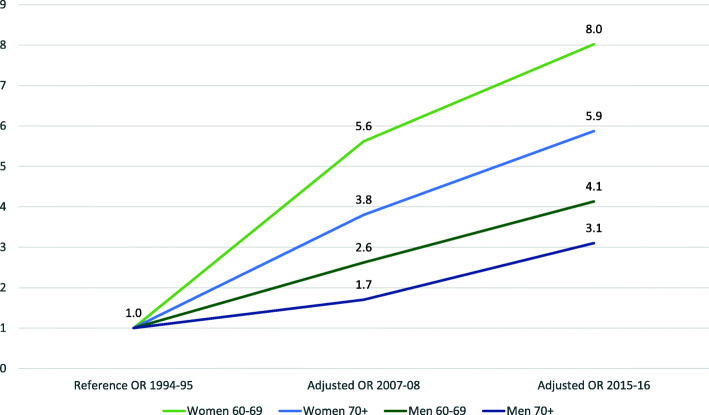
Change in adjusted OR of current drinkers reporting frequent^1^ drinking across time. ^1^Frequent drinking = AUDIT item 1, current drinkers who report to drink 2–3 times per week or more often, stratified by sex and age group

The likelihood of reporting frequent drinking increased more among women compared to men across the study period (Fig. 2).

### Trends in alcohol consumption, quantity (current drinkers)

Most participants reported their number of drinks on a typical drinking day to be 1–2 units of alcohol (Table 3).

**Table 3:** Prevalence^a^ of drinking patterns (quantity) and odds ratios (OR)^b^ across time

Age at participation	Time^c^	Moderate drinking ≤2 units on typical drinking days (drinkers only)		At-risk drinking ≥3 units/≥36 g of ethanol on typical drinking days (drinkers only)		Heavy episodic drinking (HED)^d^ (drinkers only)	
		% (N)	OR (95% CI)	% (N)	OR (95% CI)	% (N)	OR (95% CI)
**Women**							
60–69	1	84.0 (928/1105)	1	16.0 (177/928)	1	37.2 (295/793)	1
	2	84.2 (1448/1719)	1.02 (0.82–1.27)	15.8 (271/1719)	0.98 (0.79–1.22)	19.9 (342/1721)	0.43 (0.36–0.53)
	3	78.0 (1854/2377)	0.66 (0.54–0.81)	22.0 (523/2377)	1.51 (1.23–1.86)	26.9 (646/2400)	0.70 (0.59–0.84)
70+	1	91.6 (767/837)	1	8.4 (70/837)	1	–	
	2	94.6 (720/761)	1.84 (1.21–2.80)	5.4 (41/761)	0.54 (0.36–0.83)	11.6 (88/756)	1
	3	92.6 (1182/1277)	1.41 (0.98–2.04)	7.4 (95/1277)	0.71 (0.49–1.02)	15.1 (198/1315)	1.50 (1.32–1.71)
**Men**							
60–69	1	71.6 (904/1263)	1	28.4 (359/1263)	1	66.6 (749/1125)	1
	2	59.1 (1079/1826)	0.53 (0.45–0.62)	40.9 (747/1826)	1.90 (1.61–2.24)	60.4 (1103/1825)	0.91 (0.77–1.07)
	3	56.3 (1315/2337)	0.47 (0.40–0.55)	43.7 (1022/2337)	2.13 (1.81–2.50)	65.7 (1546/2352)	1.22 (1.04–1.43)
70+	1	83.0 (703/847)	1	17.0 (144/847)	1	–	
	2	80.3 (635/791)	0.83 (0.63–1.08)	19.7 (156/791)	1.21 (0.92–1.58)	33.9 (269/793)	1
	3	75.7 (1078/1424)	0.60 (0.47–0.76)	24.3 (346/1424)	1.68 (1.32–2.14)	42.3 (614/1453)	1.87 (1.55–2.25)

^a^All age group by sex prevalence rate changes were statistically significant between 1994 and 95 and 2015–16, except for at-risk drinking in women aged 70+ years

^b^OR from Generalized Equations Models with 1994–95 as reference, stratified by age group and sex, adjusted by educational level and relationship status

^c^Time: 1 = Baseline, 1994–95, 2 = 2007–08, 3 = 2015–16

^d^HED = any drinking ≥6 units/≥72 g of ethanol in one sitting last 12 months. Only participants aged < 70 years were included in 1994–95, thus 2007–08 was set as baseline in analysis of participants aged ≥70 years

However, the prevalence of at-risk drinking (≥3 units/≥36 g of ethanol per occasion) on a typical drinking day increased significantly during the study period among women aged 60–69 years from 16 to 22%, and among men from 28 to 44% in the age groups 60–69 and from 17 to 24% among those 70+ years (*p* < 0.001 in all age groups). Men have increased at-risk drinking more than women during the study period, as can be seen in the negative change in sex differences between 1994 and 95 and 2015–16 (Table 4).

**Table 4:** Prevalence rates, sex differences^a^, and change in sex differences^b^ in drinking patterns across time

Drinking pattern	Tromsø 4 (1994–95)			Tromsø 7 (2015–16)			Change in sex difference^b^ 1994–95 versus 2015–16^c^	
	Women	Men	Multivariate adjusted sex differences OR (CI 95%)	Women	Men	Multivariate adjusted sex differences OR (CI 95%)	T4 versus T7	*P*
Abstaining								
60–69	31.6	14.5	0.38 (0.31–0.46)	9.1	5.1	0.56 (0.45–0.71)	0.18	=0.026
70+	48.2	25.5	0.37 (0.30–0.46)	23.0	10.8	0.43 (0.35–0.53)	0.06	=0.374
Frequent drinking, ≥2–3 times/week								
60–69	5.2	14.2	3.02 (2.22–4.12)	34.3	41.4	1.36 (1.21–1.53)	1.66	< 0.001
70+	4.3	12.3	3.06 (2.07–4.54)	26.9	33.6	1.36 (1.16–1.60)	1.70	< 0.001
At-risk drinking, (≥3 units/≥36 g of ethanol on typical drinking days)								
60–69	16.0	28.4	2.07 (1.69–2.54)	22.0	43.7	2.80 (2.47–3.18)	−0.73	< 0.001
70+	8.4	17.0	2.17 (1.59–2.95)	7.4	24.3	4.06 (3.18–5.17)	−1.89	< 0.001
Any HED (≥6 units/≥72 g of ethanol in one sitting) last year								
60–69	37.2	66.6	3.72 (3.03–4.57)	26.9	65.7	5.72 (5.03–6.51)	−2.00	< 0.001
70+	11.6	33.9	3.96 (2.97–5.28)	15.1	42.3	5.35 (4.35–6.57)	−1.76	=0.085

^a^Sex differences reported as odds ratios (OR), with 95% confidence intervals (CI 95%), adjusted for age, level of education and relationship status with women as references

^b^Change in sex difference: positive change indicates convergence (i.e. differences growing narrower), negative change indicates divergence. P-value for interaction term between sex and survey with 1994–95 as reference

^c^Only participants aged < 70 years were included in 1994–95, thus 2007–08 was set as baseline in analysis of participants aged ≥70 years

### Trends in heavy episodic drinking (HED)

The overall prevalence of older adults aged 60 to 70 years reporting any HED during the last year was reduced from 54% in 1994–95 to 41% in 2007–08 and to 46% in 2015–16 (*p* < 0.001). The overall prevalence of older adults aged 70+ years reporting any HED during the last year increased from 23% in 2007–08 to 26% in 2015–16 (*p* = 0.020). Men aged 60–69 years have increased any HED more than women in the same age group during the study period, as can be seen in the significant negative change in sex differences between 1994 and 95 and 2015–16 (Table 4). Although the models controlling for educational level and relationship status did not find significant differences compared to unadjusted models, a modest trend was observed towards a higher probability of reporting HED and at-risk drinking among those with higher educational level in the last survey (Table 5).

**Table 5:** Three models of the probability of reporting drinking patterns across time^a^

	Tromsø 6 (2007–08)			Tromsø 7 (2015–16)		
	Unadjusted OR (95% CI)	Model 1^b^ OR (95% CI)	Model 2^c^ OR (95% CI)	Unadjusted OR (95% CI)	Model 1^b^ OR (95% CI)	Model 2^c^ OR (95% CI)
**Women ≥ 60 years**						
Abstaining^d^	0.45 (0.40–0.50)	0.50 (0.44–0.60)	0.53 (0.46–0.60)	0.26 (0.23–0.29)	0.27 (0.25–0.31)	0.29 (0.25–0.33)
Infrequent drinking^e^	0.12 (0.10–0.15)	0.18 (0.15–0.23)	0.21 (0.16–0.26)	0.08 (0.06–0.10)	0.12 (0.09–0.15)	0.14 (0.11–0.18)
Frequent drinking^f^	5.93 (4.72–7.46)	5.48 (4.35–6.90)	4.81 (3.79–6.12)	9.06 (7.28–11.29)	8.49 (6.78–10.62)	7.19 (5.69–9.10)
Moderate drinking^g^	1.01 (0.84–1.20)	0.98 (0.82–1.17)	1.10 (0.91–1.33)	0.72 (0.62–0.85)	0.68 (0.58–0.81)	0.78 (0.66–0.93)
At-risk drinking^h^	0.99 (0.83–1.19)	0.88 (0.73–1.05)	0.81 (0.67–0.98)	1.38 (1.18–1.62)	1.41 (1.19–1.67)	1.29 (1.08–1.55)
Any HED last year^i^	0.42 (0.35–0.51)	0.44 (0.37–0.53)	0.43 (0.36–0.53)	0.62 (0.52–0.74)	0.68 (0.57–0.81)	0.68 (0.57–0.81)
**Men ≥ 60 years**						
Abstaining^d^	0.51 (0.44–0.60)	0.53 (0.45–0.63)	0.59 (0.49–0.70)	0.35 (0.30–0.41)	0.33 (0.28–0.39)	0.36 (0.31–0.43)
Infrequent drinking^e^	0.41 (0.36–0.48)	0.43 (0.37–0.50)	0.45 (0.39–0.53)	0.26 (0.23–0.30)	0.27 (0.23–0.31)	0.27 (0.23–0.31)
Frequent drinking^f^	2.43 (2.10–2.81)	2.31 (1.99–2.69)	2.22 (1.90–2.60)	3.87 (3.37–4.44)	3.88 (3.35–4.49)	3.73 (3.20–4.34)
Moderate drinking^g^	0.60 (0.53–0.69)	0.56 (0.49–0.64)	0.59 (0.51–0.67)	0.57 (0.51–0.64)	0.51 (0.45–0.58)	0.53 (0.47–0.61)
At-risk drinking^h^	1.66 (1.46–1.88)	1.66 (1.45–1.90)	1.62 (1.41–1.87)	1.75 (1.55–1.97)	2.07 (1.82–2.36)	2.03 (1.77–2.32)
Any HED last year^i^	0.77 (0.66–0.90)	0.82 (0.70–0.96)	0.83 (0.71–0.97)	0.96 (0.83–1.12)	1.22 (1.04–1.42)	1.23 (1.05–1.44)

^a^Odds ratios (OR) with 95% confidence intervals (CI 95%), from Generalized Equations Models with 1994–95 as reference, stratified by sex

^b^Adjusted for age and educational level (low/ high)

^c^Adjusted for age, educational level (low/ high) and relationship status (living alone/with a partner)

^d^Teetotaller or not drinking alcohol last 12 months; ^e^ < 2 times/week; ^f^ ≥ 2–3 times/week; ^g^ ≤ 2 units on typical drinking days; ^h^ ≥ 3 units/≥36 g of ethanol on typical drinking days; ^i^ ≥ 6 units/≥72 g of ethanol in one sitting

## Discussion

### Changing drinking patterns

We identified a significant increase in the proportion of current drinkers among older adults in Norway between 1994 and 2016. Infrequent drinking is markedly reduced, and more among women than among men. Correspondingly, we found a significant increase in frequent drinking among current drinkers, larger among women than among men. The proportion who reported an increased quantity of alcohol consumed on typical drinking days increased during the study period. Any HED during the last year was modestly reduced in those aged 60–69 years, whereas a modest increase in the prevalence of any HED was found in those aged 70 years and over. Any HED last year and at-risk drinking on typical drinking days remained the alcohol measures with the largest discrepancy between men and women.

Our finding of only 7% men and 15% women reporting abstinence in 2015–16 is in contrast to the findings by Nuevo et al. (2015) from 14 European countries, where an average of 55% abstainers was found among older adults over 60 years [13], the same prevalence as reported among US older adults [37]. It is, however, in line with epidemiologic studies from Norway and other Nordic countries with an observed prevalence of abstinence between 7 and 23%, depending on age group and sex [18, 38–40].

The total prevalence of 27% among female and 36% among male older adults who reported frequent drinking in 2015–16 is considerably higher than the prevalence of 14–16% among younger adults (aged 15–59) who report frequent drinking in Norway [38]. The increase in frequent drinking was also more extensive among women during the study period, which indicate that women’s drinking patterns are approaching those of men. This is well in line with other epidemiological findings across Europe [14, 15, 20, 41], but the sex differences we found in frequent drinking in the latest survey are considerably smaller than observed in other European countries [13, 17, 21]. The findings are in accordance with recent population surveys from the Nordic countries [18, 34, 38, 40]. General societal changes over the last decades, such as an increase in women’s rights, increased work participation for women and improvement of socioeconomic status relative to men’s, may partly explain the reduced sex differences in frequent drinking [17, 19].

The prevalence of frequent drinking in the latest survey is higher than reported in several other studies [7, 13, 14, 34]. All participants in our study live in a medium sized Norwegian city, whereas other studies have included older adults from both rural and urban areas. People living in urban areas drink more than those in rural areas [38], which can partly explain our findings. Although higher educational level has been found to be associated with more frequent drinking [13, 15], our models that adjusted for this covariate did not significantly change the probability of reporting frequent drinking. Our finding of more frequent drinking among older adults stands in contrast to the observed decrease in total alcohol consumption in Norway since 2008 [16, 38], suggesting a shift in alcohol consumption from younger to older regular drinkers.

Some studies have found that the more often people drink, the more often they drink to intoxication [42, 43], and there is a strong and consistent correlation between mean consumption in a population and the proportion of at-risk drinkers [44]. This could partly explain our parallel findings of more frequent drinking and drinking larger quantities. Our study reports an increase in at-risk drinking in both women and men aged 60–69 years, and in men aged 70 years and older. This is in contrast to other recent findings from Nordic countries, where this drinking behaviour was found to be relatively stable since 2000 [38, 40]. Gell et al. found large variations in excessive drinking among older adults both between and within countries, in a comparative study of drinking patterns across developed countries, including Europe, the US and Australia; from 4 to 36% (defined as ≥2 units among women and ≥ 3 units among men) [15].

Binging is considered to be most harmful in old age [28, 37], and our study shows that 46% of participants between 60 and 70 years reported HED on at least one occasion last year. This prevalence of HED was larger than observed in other European countries [5, 14, 45]. Several of the studies on alcohol consumption in older adults and findings reported in systematic reviews are, however, based on older data. A more recent study from New Zealand found that 58% of men and 20% of women among community dwelling older adults aged 55–70 years reported HED at least once yearly using the AUDIT-C, which is in line with our findings [28]. Another recent study from Norway reported an increase in any HED, from 17% in 1985 to 30% in 2016–17 [38]. A comparative study from the Nordic countries, also reported increased prevalence of HED among older adults since 2000 [40]. At-risk drinking (≥3 units/≥36 g of ethanol) on typical drinking days and any HED (≥6 units/≥72 g of ethanol in one sitting) last year remained the alcohol measures with the largest discrepancies between men and women across the study period. Biological factors, including greater sensitivity to adverse health effects due to binge drinking among women, may explain part of the sex differences observed in these alcohol measures [11, 14].

### Alcohol policy and societal changes

The primary objective of Norwegian alcohol policy has been to minimize alcohol-related health and social problems at the population level [44]. During the twentieth century, Norway has probably had one of the most restrictive alcohol policies in Europe with high prices and restricted availability, and in 2000 the level of alcohol consumption in Norway was one of the lowest in Europe [46]. The key features of current older adults in Norway, as in many other Western countries, are a higher educational level compared to previous generations, higher income, changing gender roles and a stronger focus on individualism, self-realisation and pleasure [47–49].

Changing alcohol habits have been suggested to represent a cohort effect from the “baby boomers” (those born between 1946 and 1964), who had higher exposure to alcohol in their youth and tended to be more tolerant about substance use than earlier generations [5]. More liberal attitudes towards alcohol among elderly people in Europe have been reported [14, 47, 49], as well as scepticism about the health risks of alcohol and even the view that not drinking alcohol could be negative for health [11, 47, 50]. The first generation of the baby boomers turned 65 years in 2011, hence, not all changes observed in the present study can be explained by such a cohort effect. It has also been suggested that drinking habits are “contagious” [44, 51], suggesting that increased alcohol consumption among younger cohorts of older adults may affect drinking habits in older cohorts. Furthermore, Norwegian senior citizens have greater financial security, better health and welfare schemes, less social inequality and more gender equality than in many other European countries [49]. These characteristics of societal and cultural differences may help explain the changing drinking patterns among older adults in Norway.

Importantly, the supply of cheaper alcoholic beverages through cross-border and international tax-free shopping has increased in recent decades, as has the number of alcohol outlets in Norway, and the sales of 3 litre wine cartons have become mainstream [16]. Previous findings of European levels of daily drinking have shown a north-south gradient with relatively higher consumption of wine in Southern Europe compared to Northern Europe, but fewer monthly binge drinking sessions [14]. Over the last two decades, total alcohol consumption in Norway has changed with increased wine sales and decreased beer and spirits sales [16]. It has been suggested that the drinking culture from Mediterranean countries, where many Norwegians take their vacations and where many seniors have “second homes”, may have been adopted [16, 18]. However, our findings of both increased frequent drinking, in combination with preserved habits of bingeing, suggest the emergence of new drinking patterns among the Norwegian older adults with a possible combination of northern European and southern European drinking traditions.

Our findings support and extend accumulating evidence that sex differences in frequent alcohol consumption are decreasing [15, 17, 18, 20, 21], even in the oldest age groups, possibly suggesting shifting social norms surrounding gender and alcohol consumption. Holmila and Raitasalo (2005) have proposed social mechanisms mediating changes in women’s drinking, including the stress caused by women’s dual roles, the mimicking of male drinking patterns, changes in male-female drinking companionship, and changes in alcohol’s position as a symbol of gender roles [22].

### Clinical implications

The findings of this study may be particularly important for general practitioners and other health professionals. Important interventions, such as health advice on the increased risk of falls, accidents and confusion due to alcohol use, may not be reaching older adults as a result of symptom misinterpretation and a lack of key skills among health professionals in identifying and managing risky alcohol use in elderly people [10, 11]. Raising public awareness of the substantial changes in alcohol habits among older adults is therefore important.

### Main strengths and limitations

The primary strengths of the population based Tromsø Study are the high number of participants from the same geographical area, the repeated survey design and the high rates of attendance, ensuring a high degree of representation. However, the proportion of participants in the oldest age group in our study was relatively low and may therefore be less representative of the general population. Since there has been few studies conducted including the oldest age group (70 years and older), our findings may nevertheless contribute to the evidence on alcohol consumption among older adults.

The Tromsø Study is based on self-reporting questionnaires, and because adults tend to underestimate their own alcohol consumption [52], there may be an underestimation of the alcohol consumption level. Furthermore, older people are even more likely to underreport alcohol use [53–55]. However, more liberal attitudes towards alcohol use in old age, including among older women, may have reduced stigma and shame in the last survey, and this may have contributed to less underreporting. In addition, variation in how questions were asked in the three surveys makes it necessary to exercise caution when interpreting the comparison across time. Open-ended questions about frequency and volume (as in Tromsø 4), without categorical response options (as in Tromsø 6 and Tromsø 7), may have increased the tendency to underestimate self-reported alcohol consumption. However, the significant findings on prevalence and sex differences in the two last surveys are based on identical questions.

As in general population surveys elsewhere [56], the participation rate in the Tromsø Study has declined [31], especially among participants aged 70 years and older. Alcohol misuse, abstaining from alcohol, and mental distress are moderately associated with non-participation in population surveys [57, 58]. However, in a comparable study from another county in Norway, this association weakened when controlling for other variables [56]. Nevertheless, the underrepresentation of people with high alcohol consumption, abstainers and people with poor mental health should be taken into consideration when interpreting results from population-based health surveys.

As the Tromsø Study is based in the seventh largest Norwegian city with relatively few immigrants, it is limited with regard to ethnic diversity. The generalizability of results may therefore be limited to Caucasian populations that are similar to older adults of Norwegian descent. Furthermore, since the sample does not include rural living older adults the generalizability in prevalence rates of alcohol consumption may be restricted to urban living older adults.

## Conclusions

Among older adults in Norway, alcohol consumption has increased considerably from 1996 to 2016. Compared to previous generations, the new generation of older adults drinks more frequently and consumes larger quantities on typical drinking days, while the prevalence of heavy episode drinking remains stable. The gap between women and men in frequent drinking has been markedly narrowed, suggesting that women’s drinking patterns are approaching those of men. Even though overall drinking has increased, the changes are not necessarily connected to alcohol-related harm per se. Women and older adults are, however, particularly susceptible to the harmful effects of alcohol, which may imply that a change in governmental strategies and alcohol policy to influence alcohol-related health behaviours to more targeted interventions for elderly people is needed.

## Supplementary Information

**Additional file 1: Figure S1.** Alcohol consumption during the last year in three surveys from the Tromsø Study^1^ (Additional file 2, .pptx) ^1^Tromsø 4 = 1994–95, Tromsø 6 = 2007–08, and Tromsø 7 = 2015–16. From crude data.

**Additional file 2: Table S1.** Classification of alcohol outcome measures in Tromsø 4, Tromsø 6, and Tromsø 7.

## Data Availability

The data that support the findings of this study are available from (http://tromsoundersokelsen.uit.no/tromso/) but restrictions apply to the availability of these data, which were used under license for the current study, and so are not publicly available. Research groups may apply for access to the data (see instructions on the website).

## Abbreviations

AUDIT-C: Alcohol Use Disorders Identification Test-Consumption
CI: Confidence interval
GEE: Generalized estimating eqs.
HED: Heavy episodic drinking. Drinking ≥6 units of alcohol/≥72 g of ethanol in one sitting last 12 months
OR: Odds ratio
