# Supplementary material for: The changing alcohol drinking patterns among older adults show that women are closing the gender gap in more frequent drinking: the Tromsø study, 1994–2016
Source: Subst Abuse Treat Prev Policy. 2021 May 26;16:45. doi: 10.1186/s13011-021-00376-9 (PMC8152329; doi:10.1186/s13011-021-00376-9)
Supplement: Supplementary file 1 — Additional file 1: Figure S1. Alcohol consumption during the last year in three surveys from the Tromsø Study1 (Additional file 2, .pptx) 1Tromsø 4 = 1994–95, Tromsø 6 = 2007–08, and Tromsø 7 = 2015–16. From crude data. [file 13011_2021_376_MOESM1_ESM.ppt]

## Slide 1
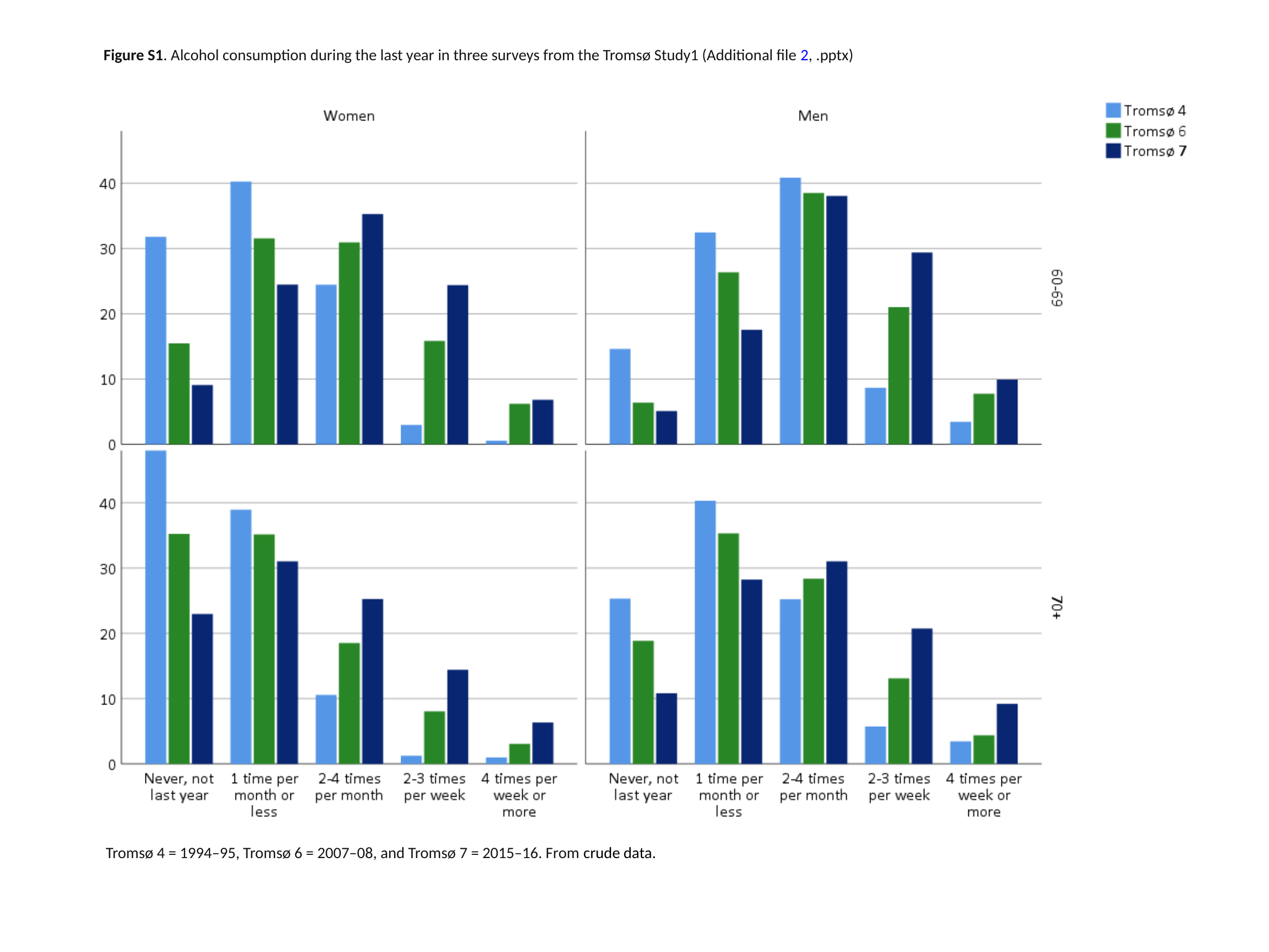

Figure S1. Alcohol consumption during the last year in three surveys from the Tromsø Study1 (Additional file 2, .pptx)
Tromsø 4 = 1994–95, Tromsø 6 = 2007–08, and Tromsø 7 = 2015–16. From crude data.
