# Supplementary material for: The changing alcohol drinking patterns among older adults show that women are closing the gender gap in more frequent drinking: the Tromsø study, 1994–2016
Source: Subst Abuse Treat Prev Policy. 2021 May 26;16:45. doi: 10.1186/s13011-021-00376-9 (PMC8152329; doi:10.1186/s13011-021-00376-9)
Supplement: Supplementary file 2 — Additional file 2: Table S1. Classification of alcohol outcome measures in Tromsø 4, Tromsø 6, and Tromsø 7. [file 13011_2021_376_MOESM2_ESM.docx]

**Supplementary Table 1** Classification of alcohol outcome measures in Tromsø 4, Tromsø 6, and Tromsø 7.

| **Outcomes** | **Tromsø 4 (1994-95)** | **Tromsø 6 (2007-08) and Tromsø 7 (2015-16)** |
| --- | --- | --- |
| **Abstaining**  **(AUDIT-C, item 1)** | **Q1:** Are you a teetotaler?  (Yes or No response)  Abstaining = yes | How often do you drink alcohol?  (Never, Monthly or less, 2-4 times a month, 2-3 times a week, 4 times a week or more)  Abstaining = Never |
| **Alcohol consumption, frequency^a^**  **(AUDIT-C, item 1)** | **Q2:** How many times a month do you normally drink alcohol? Do not count low-alcohol beer (2.5% ethanol). Put 0 if less than once a month  (Open response)  **Q2:** Mean: 2.62 (SD 4.80) | How often do you drink alcohol?  (Never, Monthly or less, 2-4 times a month, 2-3 times a week, 4 times a week or more)  Dichotomized as “Infrequent” (2-4 times a month or less frequent) or “Frequent” (2-3 times per week or more frequent) |
| **Alcohol consumption, quantity^b^**  **(AUDIT-C, item 2)** | **Q3-5:** How many glasses of beer, wine or spirits do you usually drink in the course of two weeks?  Do not include low-alcohol beer. Write 0 if less than once a month.  (Open response)  **Q3:** Beer: Mean 0.93 (SD 3.33)  **Q4:** Wine: Mean 1.11 (SD 2.93)  **Q5:** Spirits: Mean 1.36 (SD 3.15). | How many units of alcohol (one beer, a glass of wine, or a drink/spirits) do you usually drink when you drink alcohol?  (1-2, 3-4, 5-6, 7-9, 10 or more)  Dichotomized as “1-2” = ”Moderate drinking” = ≤2 units of alcohol / ≤24 grams of ethanol, or “3-4”, “5-6”, “7-9”, “10 or more” = ”At-risk drinking” = ≥3 units of alcohol/≥36 grams of ethanol on typical drinking days |
| **Heavy episodic drinking (HED)^c^**  **(AUDIT-C, item 3)** | **Q6:** Approximately how often during the past 12 months have you drunk alcohol corresponding to at least five bottles of beer, a bottle of wine or a quarter of bottle of spirits?  (Not at all the past year, A few times, Once or twice a month, Once or twice a week, Three or more times a week”) | How often do you drink 6 units of alcohol or more in one occasion?  One unit of alcohol = 12 grams of ethanol = one beer, one glass of wine or one glass of spirits?  (Never, Less than monthly, Monthly, Weekly, Daily or almost daily) |

^a^The categorization according to the AUDIT item 1 were estimated as follows:

- “Never” = responded “Yes” to the question “Are you a teetotaler”
- “Monthly or less” (responded 0 or 1 time to the question “How many times a month do you normally drink alcohol” but responded “no” to the question “Are you a teetotaler”)
- “2-4 times a month” (responded 2-7 times to the question “How many times a month do you normally drink alcohol”)
- “2-3 times a week” (responded 8-15 times to the question “How many times a month do you normally drink alcohol”)
- “4 times a week or more” (responded ≥16 times to the question “How many times a month do you normally drink alcohol”)

^b^The categorization according to the AUDIT item 2 were estimated as follows:

- The monthly number of alcoholic units consumed was estimated by adding together the beverage units reported in a usual two-week period, multiplied by two (to have monthly consumption).
- To estimate number of alcohol units on typical drinking days, the overall monthly consumption was divided by the reported monthly frequency of alcohol consumption.
- The alcohol quantity variable was dichotomized to “moderate” (≤2 units/≤24 grams of ethanol) or “at-risk” (≥3 units/≥36 grams of ethanol) drinking on typical drinking days.

^c^The categorization according to the AUDIT item 3 were estimated as follows:

Heavy episodic drinking estimates ≥72 grams of ethanol in Norway (only persons <70 years were given this question in Tromsø 4).

Response alternatives: “Not at all the past year” (53.7%), “A few times” (31.5%), “Once or twice a month” (9.7%), “Once or twice a week” (4.3%), “Three or more times a week” (0.8%)

- Response alternatives in the surveys were dichotomized in this study to “Never” or “Ever” HED during the past year
